# Micheliolide provides protection of mice against *Staphylococcus aureus* and MRSA infection by down-regulating inflammatory response

**DOI:** 10.1038/srep41964

**Published:** 2017-02-06

**Authors:** Xinru Jiang, Yuli Wang, Yifei Qin, Weigang He, Adel Benlahrech, Qingwen Zhang, Xin Jiang, Zhenhui Lu, Guang Ji, Yuejuan Zheng

**Affiliations:** 1 Department of Immunology and Microbiology, Shanghai University of Traditional Chinese Medicine, Shanghai 201203, China; 2 School of Clinical Medicine, Shaanxi University of Chinese Medicine, Xi xian New District, Shaanxi 712046, China; 3 MRC Human Immunology Unit, Weatherall Institute of Molecular Medicine, Nuffield Department of Medicine, University of Oxford, Oxford OX3 9DS, UK; 4 Department of Respiration, Longhua Hospital, Shanghai University of Traditional Chinese Medicine, Shanghai 200032, China; 5 Institute of Digestive Diseases, China-Canada Center of Research for Digestive Diseases (ccCRDD), Longhua Hospital, Shanghai University of Traditional Chinese Medicine, Shanghai 200032, China

## Abstract

A major obstacle to therapy in intensive care units is sepsis caused by severe infection. In recent years gram-positive (G^+^) bacteria, most commonly staphylococci, are thought to be the main pathogens. Micheliolide (MCL) was demonstrated to provide a therapeutic role in rheumatoid arthritis, inflammatory intestinal disease, colitis-associated cancer, and lipopolysaccharide (LPS, the main component of G^−^ bacterial cell wall) induced septic shock. We proved here that MCL played an anti-inflammatory role in *Staphylococcus aureus (S. aureus*) and methicillin-resistant *S. aureus* (MRSA) induced peritonitis. It inhibited the expression of inflammatory cytokines and chemokines in macrophages and dendritic cells upon stimulation with peptidoglycan (PGN, the main cell wall composition of G^+^ bacteria). PI3K/Akt and NF-κB pathways account for the anti-inflammatory role of MCL after PGN stimulation. MCL reduced IL-6 secretion through down-regulating NF-κB activation and improved the survival status in mice challenged with a lethal dose of *S. aureus*. In MRSA infection mouse model, MCL down-regulated the expression of IL-6, TNF-α, MCP-1/CCL2 and IFN-γ in sera, and ameliorated the organ damage of liver and kidney. In conclusion, MCL can help maintain immune equilibrium and decrease PGN, *S. aureus* and MRSA-triggered inflammatory response. These provide the rationality for the potential usage of MCL in sepsis caused by G^+^ bacteria (e.g., *S. aureus*) and antibiotic-resistant bacteria (e.g., MRSA).

Sepsis is still the leading cause of death in U.S. hospitals, characterized by pathogenic infection and systemic inflammatory response syndrome (SIRS)^1^. Severe sepsis may cause acute organ dysfunction^2^, and affect up to 19 million individuals each year worldwide^3^. The mortality rate remains high despite progress in treatment^4^. During the last decade, mortality from sepsis was increased by 17%^5^. Recently, statistical data suggested that the mortality rate of patients with sepsis ranged from 34 to 52%^6^. Gram-negative (G^−^) bacteria were usually the predominant organisms causing sepsis, but in recent years gram-positive (G^+^) bacteria are thought to be the main pathogens causing approximately 57% of cases of sepsis^7^,^8^. Among the G^+^ bacteria associated with sepsis, *Staphylococcus aureus (S. aureus*) is the most frequent organism with the highest mortality^9^. Although all G^−^ bacteria had decreased mortality hazards, methicillin-resistant *S. aureus* (MRSA) had the highest mortality hazard of 1.38 (1.33–1.44) of G^+^ organisms^9^. Even though early use of antibiotics increased the survival chances of patients with sepsis, the patients still face a higher risk of death due to uncontrolled inflammation and drug resistance^10^,^11^.

The pathogenesis of sepsis is associated with the excessive and harmful production of cytokines. The overwhelming production of inflammatory cytokines was shown to result in multiple organ dysfunction with subsequent organ failure^12^. Toll-like receptor (TLR) family and nucleotide-binding oligomerization domain-containing proteins (NOD) family are two main receptor families which recognize pathogen-associated molecular patterns (PAMPs) and elicit inflammatory immune response^13^. In contrast to TLRs, which recognize microbial ligands at the levels of cellular surface or endosomes, the predominant member of NOD family, NOD1, NOD2 and cryopyrin, sense bacterial products in the cytosol to provide another microbial surveillance. NOD 1 recognizes PGN from G^−^ bacteria and NOD2 recognizes PGN from both G^+^ and G^−^ bacteria^14^.

During *S. aureus* infection, the bacteria activate TLR 2/6 heterodimers, NOD2 and other receptors through the ligation of their corresponding ligands, e.g., peptidoglycan (PGN), the main component of the G^+^ bacterial cell wall and bacterial lipoprotein. These are thought to be the major contributors to the production of inflammatory cytokines and the imbalance of homeostasis^15^. Upon PGN stimulation, TLR2 recruits myeloid differentiation factor 88 (MyD88) to activate the downstream signaling. TLR2-MyD88 signaling complex recruits interleukin-1 receptor-associated kinases (IRAKs), tumor necrosis factor (TNF) receptor-associated factor 6 (TRAF6), and transforming growth factor (TGF)-activated kinase 1 (TAK1) to mediate mitogen-activated protein kinases (MAPKs) and nuclear factor κB (NF-κB) activation^16^, which account for the production of pro-inflammatory cytokines, such as interleukin 6 (IL-6), TNF-α, IL-1β, chemokines, interferons, and anti-inflammatory cytokine IL-10^17^. Phosphatidylinositol (PI) 3-kinase (PI3K) signaling pathway also contributes to TLR2-activated immune response, promoting IL-10 but inhibiting IL-12 expression in immune cells^18^,^19^. Besides recognition by TLR2/6, PGN can also be digested by lysozyme in macrophages and muramyl dipeptide (MDP), a conserved component in bacterial PGN is recognized by intracellular sensor NOD2 or cryopyrin to elicit immune response^20^. After ligation, NOD2 becomes oligomerized and recruits RIP-like interacting CLARP kinase (RICK), which activates the IKK complex by ubiquitinylation and stimulates NF-κB activation, as well as induces the activation of MAPKs, accounting for the expression of target genes IL-6, TNF-α, monocyte chemotactic protein 1 (MCP-1)/CC-chemokine ligand 2 (CCL2), etc^13^. Cryopyrin also becomes oligomerized and elicits the maturation of IL-1β and IL-18^13^.

Currently, many useful compounds derived from natural products have been demonstrated in drug discovery process and disease therapy, such as anti-cancer, anti-infection, anti-bacterial and so on^21^,^22^. Sesquiterpene lactones (SLs) are an important category of naturally occurring plant terpenoids, the majority of which have shown significant biological properties including antimalarial, anticancer, antiviral, antibacterial, antifungal and anti-inflammatory activities^23^,^24^,^25^.

Micheliolide (MCL), a sesquiterpene lactone (Fig. 1a), is derived from *Michelia compressa* (Magnoliaceae)^26^. MCL has also been reported to be beneficial to rheumatoid arthritis^27^, inflammatory intestinal disease and colitis-associated cancer^28^. In the previous study, we recently demonstrated that MCL inhibits LPS-induced inflammatory response and protects mice from LPS induced endotoxic shock^29^. Therefore, we wondered whether MCL could play a protective role in sepsis caused by G^+^ bacteria or even antibiotic-resistant G^+^ bacteria. In this study, we demonstrated that MCL not only reduces PGN-induced inflammatory response, but also plays an anti-inflammatory and protective role in *S. aureus* and MRSA induced peritonitis mouse models.

## Results

### MCL does not promote cellular apoptosis of mouse peritoneal macrophages

**Figure 1:**
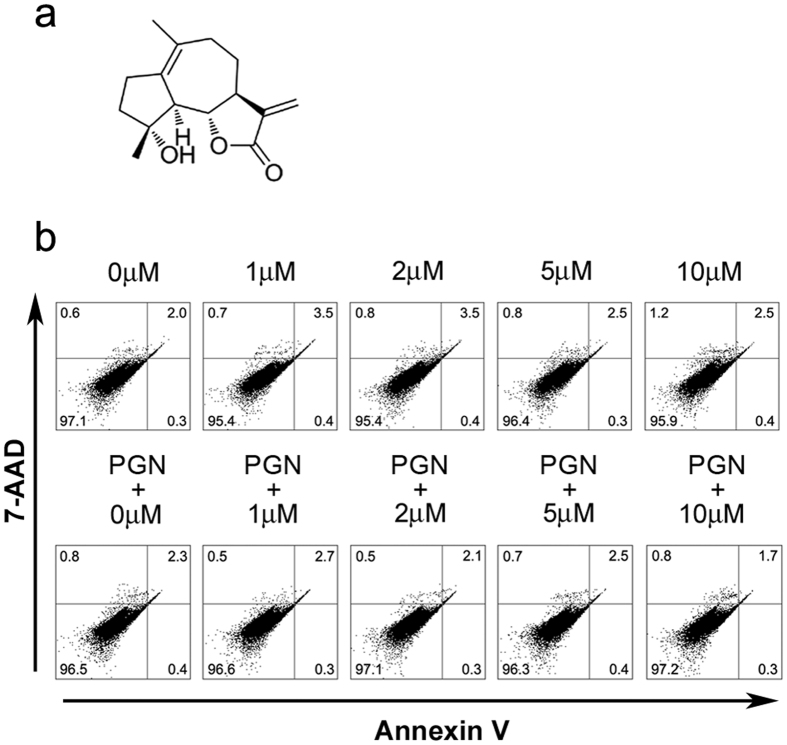
Experimental concentrations of MCL do not promote cellular apoptosis of mouse peritoneal macrophages. (**a**) Chemical structure of MCL. (**b**) Mouse primary macrophages (3.5 × 10^5^ cells/350 μL) were plated overnight, and stimulated with different concentrations of MCL in the presence or absence of PGN (25 mg/L) for 18 h. Cells were then harvested and labeled with Annexin V and 7-AAD, and analyzed by FACS. Results are representative of three independent experiments.

We have previously demonstrated that MCL has low toxicity below 10 μM and has no observed influence on cell viability of mouse macrophage cell line Raw264.7^29^. In this study, the apoptotic sensitivity of mouse peritoneal macrophages to MCL or the combination of MCL and PGN was examined. Different concentrations of MCL (1 to 10 μM) were added into the cell culture medium with or without PGN (25 mg/L). Eighteen hours later, cellular apoptosis was examined by FACS with Annexin V and 7-AAD labeling (Fig. 1b). The FACS results showed MCL did not induce apoptosis in resting or PGN-stimulated mouse peritoneal macrophages at 18 h.

### MCL reduces the production of inflammatory cytokines induced by PGN in mouse macrophages and dendritic cells (DCs)

**Figure 2:**
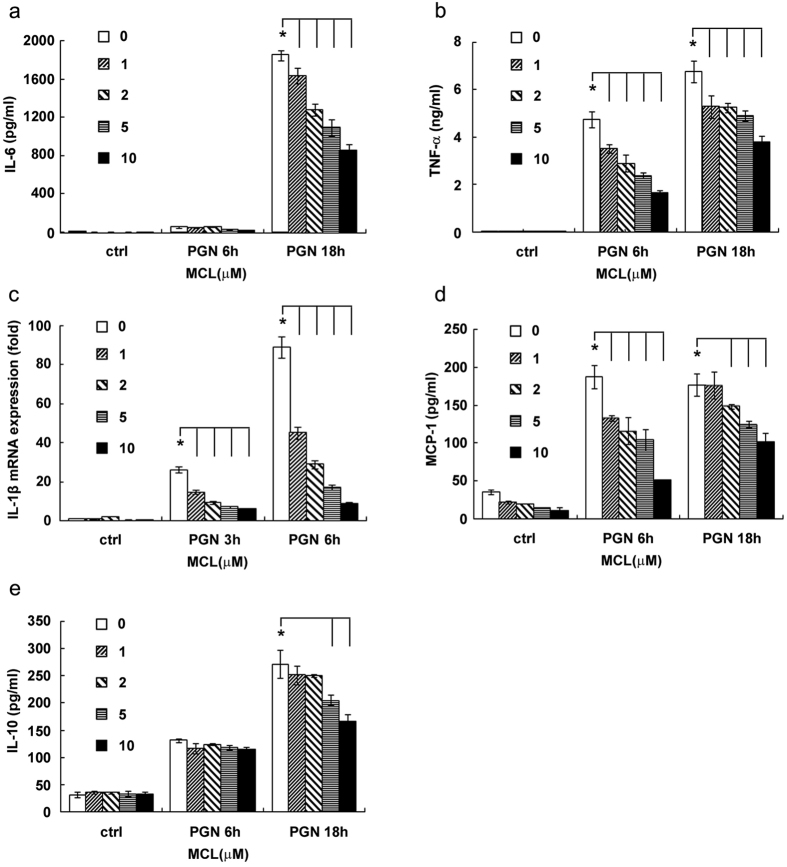
MCL inhibits PGN-induced cytokine production in Raw264.7. Raw264.7 was seeded (2 × 10^5^ cells/350 μL) in 24-well plates overnight, and stimulated by PGN (25 mg/L) and different concentrations of MCL for 3, 6 or 18 h. Cell culture supernatants were collected and the concentrations of IL-6 (**a**), TNF-α (**b**), MCP-1 (**d**) and IL-10 (**e**) were detected by ELISA. IL-1β (**c**) mRNA expression was examined by qRT-PCR. Data are shown as mean ± SD of three independent experiments. *Indicates p < 0.05.

**Figure 3:**
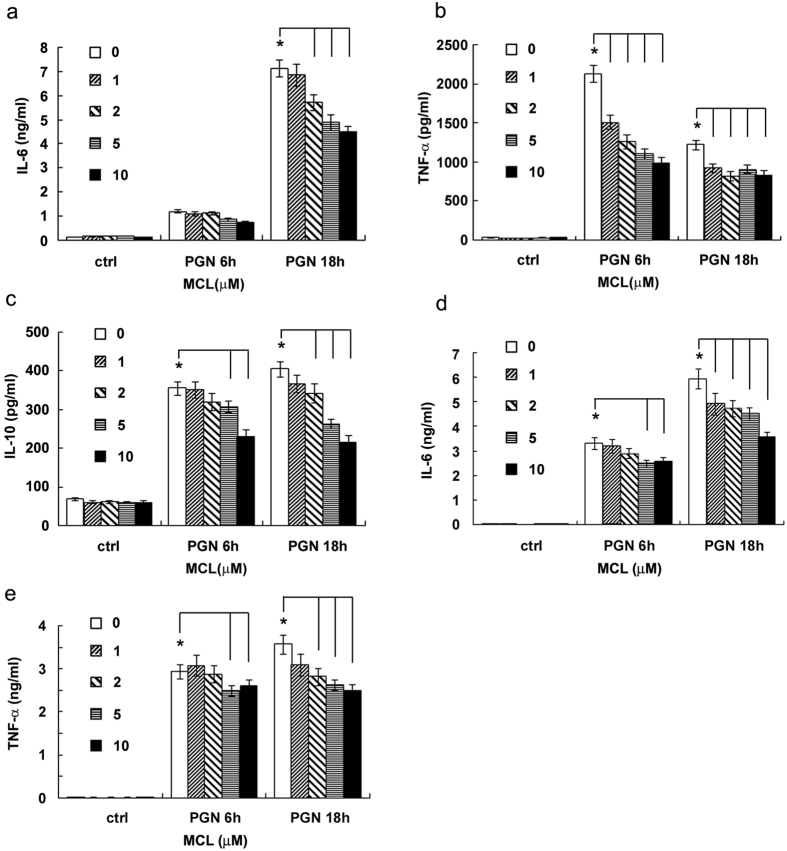
MCL decreases the production of PGN-induced IL-6, TNF-α and IL-10 in peritoneal macrophages and bone marrow-derived dendritic cells (DCs). Primary peritoneal macrophages were seeded at 3.5 × 10^5^ cells/well in 24-well plates and cultured overnight. Cells were treated with different concentrations of MCL with or without PGN (25 mg/L) for 6 or 18 h. The concentrations of IL-6 (**a**), TNF-α (**b**) and IL-10 (**c**) were measured in the supernatants by ELISA. Mouse DCs (1.5 × 10^5^ cells/300 μL) were induced as indicated and stimulated with PGN (25 mg/L) and MCL. The concentrations of IL-6 (**d**) and TNF-α (**e**) were measured by ELISA. Data are shown as mean ± SD of three independent experiments. *Indicates p < 0.05.

Both macrophages and DCs are antigen-presenting cells which produce inflammatory cytokines upon activation. They play pivotal roles in the regulation of inflammatory response and in the link between innate and adaptive immune response. In order to explore the anti-inflammatory role of MCL in the infection of G^+^ bacteria, mouse macrophage cell line Raw264.7 was stimulated simultaneously with PGN and different concentrations of MCL (1 to 10 μM). The expression of cytokines was detected by ELISA, and the mRNA level of IL-1β was assessed by qRT-PCR. Results shown in Fig. 2 indicated that MCL treatment suppressed PGN-triggered expression of inflammatory mediators, including IL-6, TNF-α, IL-1β, MCP-1 as well as the anti-inflammatory cytokine IL-10 in Raw264.7 (Fig. 2). A similar result was obtained using mouse primary peritoneal macrophages (Fig. 3a–c). Further, we examined the anti-inflammatory role of MCL in PGN-stimulated bone marrow-derived DCs (BMDCs). The results showed that MCL also suppressed IL-6 and TNF-α production in BMDCs upon stimulation with PGN (Fig. 3d,e). So, MCL was found to inhibit the production of inflammatory cytokines in response to PGN in macrophages and in DCs as well.

### MCL decreases PGN-induced inflammatory cytokine expression in human THP-1

**Figure 4:**
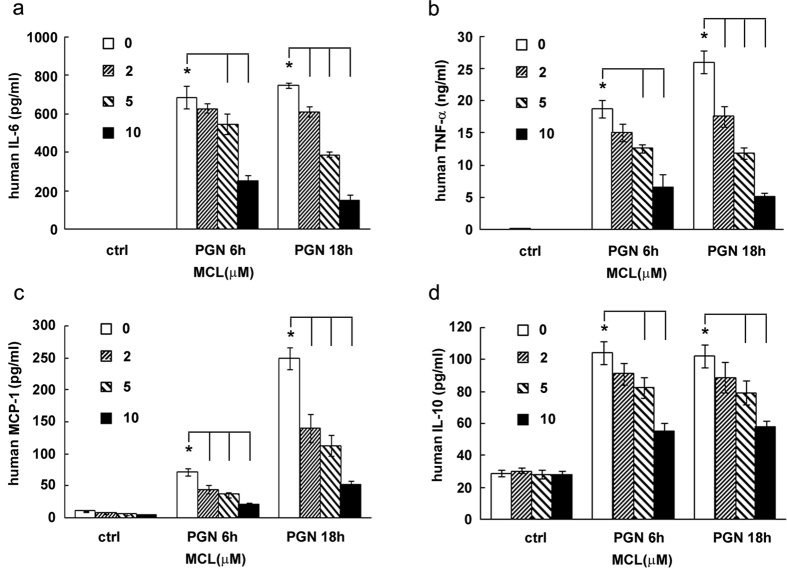
MCL decreases the production of inflammatory mediators in the human monocytic cell line THP-1. THP-1 cells were seeded in 24-well plates (1.8 × 10^5^ cells/400 μL) in the presence of phorbol-12-myristate-13-acetate (PMA, 10 ng/mL) overnight. Cells were treated with different concentrations of MCL in the presence or absence of PGN (25 mg/L) for 6 or 18 h. Secretion of IL-6 (**a**), TNF-α (**b**), MCP-1 (**c**) and IL-10 (**d**) was detected by ELISA. Data are shown as mean ± SD of three independent experiments. *Indicates p < 0.05.

We sought to examine the effects of PGN on human cells. We investigated the role of MCL on PGN-stimulated human monocytic cell line THP-1. As shown in Fig. 4, MCL decreased the expression of pro-inflammatory cytokine IL-6, TNF-α, chemokine MCP-1 and the anti-inflammatory IL-10 in THP-1. The results suggested that MCL reduced inflammatory mediator expression in cells with a human origin.

### MCL inhibits PGN-induced inflammatory response via down-regulation of NF-κB and PI3K/Akt pathways

After ligation of PGN, TLR2 or NOD2 activates the downstream adaptor MyD88 or RICK, respectively, eliciting the activation of the commonly used downstream signaling pathways, including extracellular signal regulated kinase 1/2 (ERK1/2), c-Jun N-terminal kinase (JNK), and p38 MAPK pathways, NF-κB pathway and PI3K/Akt signaling pathway^13^,^30^, accounting for the production of the downstream cytokines.

**Figure 5:**
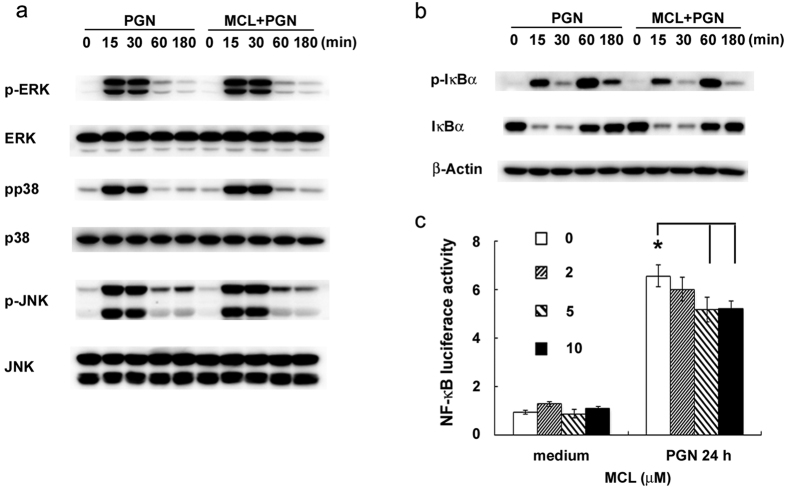
MCL inhibits PGN-induced activation of NF-κB pathway. Mouse peritoneal macrophages (1 × 10^6^ cells/well) were plated overnight, and then stimulated with PGN (25 mg/L) in the presence or absence of MCL (10 μM) for different time periods. (**a**) The phosphorylation of ERK, JNK, p38 MAPK were examined by Western blot, with the reference of the corresponding total proteins. (**b**) The expression of phospho-IκBα, total IκBα and β-Actin were examined. The results were representative of three independent experiments. (**c**) Raw264.7 cells were cotransfected with pRL-TK-Renilla-luciferase plasmid and NF-κB luciferase reporter plasmid for 30 h. Cells were treated with PGN (25 mg/L) for a further 24 h, and luciferase activities were measured. The NF-κB luciferase activities are presented as fold increase. Data are shown as mean ± SD of at least three independent experiments. *Indicates p < 0.05.

Through the screening of activation of different signaling pathways, we found that MCL did not inhibit PGN-induced ERK, JNK, and p38 MAPKs activation (Fig. 5a). NF-κB pathway is essential for inflammatory cytokine or chemokine production and the pathogenesis in some inflammatory diseases. To further validate the regulatory role of MCL on NF-κB pathway, Western blot and luciferase reporter gene assay were carried out. PGN-induced phosphorylation of IκBα (Ser32/36) in mouse primary peritoneal macrophages was down-regulated by MCL treatment (Fig. 5b). Correspondingly, MCL decreased PGN-induced activity of NF-κB luciferase reporter gene in Raw264.7 cell line by co-transfection of NF-κB luciferase reporter plasmid and pRL-TK-*Renilla*-luciferase plasmid. Thus, MCL inhibits PGN-induced NF-κB activation.

**Figure 6:**
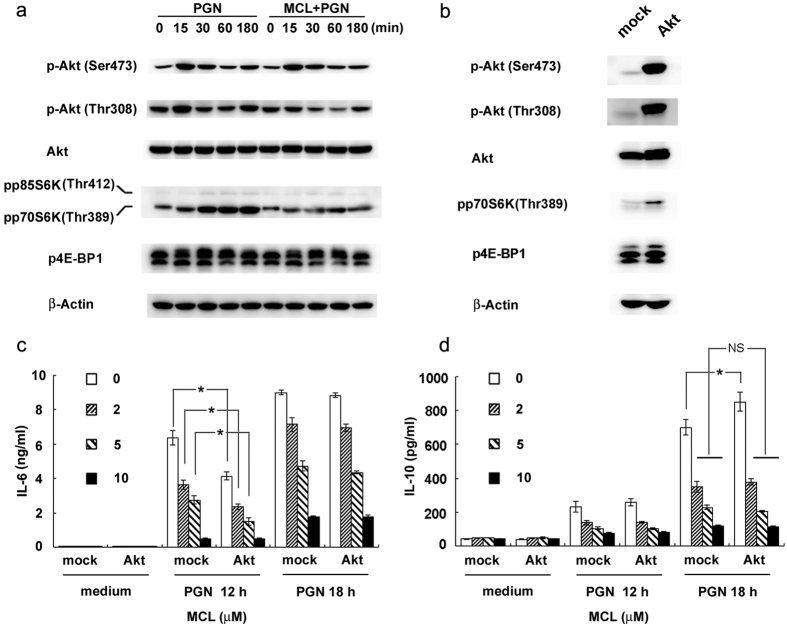
MCL inhibits PGN-induced activation of PI3K/Akt pathway, accounting for the decreased expression of IL-10. Mouse peritoneal macrophages were plated overnight and stimulated with PGN (25 mg/L) with or without MCL (10 μM) for different time courses. (**a**) The phosphorylation of Akt (Ser473 or Thr308), p70S6K (Thr389) and 4E-BP1 (Thr37/46) were examined by Western blot, with the reference of total Akt and β-Actin. (**b**–**d**) Raw264.7 cells were transfected with empty plasmid (mock) and Akt-expressing plasmid (Myr-Akt-HA), separately. The phosphorylation of Akt (Ser473 or Thr308), p70S6K (Thr389), 4E-BP1 (Thr37/46), total Akt and β-Actin were examined by Western blot. Thirty-six hours after transfection, cells were stimulated by PGN (25 mg/L) and different concentrations of MCL for the indicated time periods. Secretion of IL-6 or IL-10 in the supernatants was examined using ELISA. Similar results were obtained in three independent experiments. Data are shown as mean ± SD of three independent experiments; *p < 0.05.

Phosphorylation of Akt at Thr308 or Ser473 is considered as a marker of PI3K/Akt pathway activation. The results showed that MCL suppressed phosphorylation of Akt (Thr308) (Fig. 6a) following PGN stimulation in mouse primary peritoneal macrophages. Mammalian target of rapamycin (mTOR) is an important serine/threonine protein kinase downstream of PI3K/Akt^31^. Akt is phosphorylated by PI3K activation and promotes phosphorylation of mTOR, enhancing translation initiation partially by phosphorylation of its main targets, eukaryotic initiation factor 4E-binding protein 1/2 (4E-BP1/2) and the proteins of p70S6 kinase (p70S6K)^32^. The phosphorylation of the translational repressor 4E-BP1 by mTOR abrogates its ability to bind the initiation factor eIF4E and inhibits the transcription of target genes^33^. The production of IL-10 in mouse BMDCs or human monocyte-derived DCs was substantially diminished when cells were pretreated with rapamycin (inhibitor of mTOR) before LPS stimulation^34^. As Fig. 6a shown, MCL inhibited PGN-triggered phosphorylation of p70S6K and 4E-BP1.

To explore the exact role of PI3K/Akt pathway contributing to the immunomodulatory effect of MCL upon PGN stimulation, Raw264.7 cells were transfected with empty plasmid (mock) or Akt expressing plasmid (Myr-Akt-HA). After 36 h, cells were stimulated with PGN and different concentrations of MCL. Phosphorylation of Akt (Ser473 or Thr308), p70S6K and 4E-BP1 was enhanced by Akt over-expression (Fig. 6b). PGN-mediated IL-6 secretion in the supernatants decreased with the activation of PI3K/Akt pathway at 12 h. MCL decreased PGN-induced IL-6 expression in a dose-dependent manner, but its incubation didn’t prevent the decreased expression of IL-6 caused by Akt over-expression and activation (Fig. 6c), which implied a PI3K/Akt-independent role of MCL on PGN-induced IL-6 expression. On the contrary, PGN-stimulated IL-10 expression was enhanced by Akt over-expression and activation at 18 h (Fig. 6d). What’s more, MCL-inhibited activation of PI3K/Akt pathway accounts for the retractile expression of IL-10 enhanced by Akt over-expression/activation in PGN signaling (Fig. 6d). Thus, MCL inhibited PGN-induced IL-10 expression at least partially through PI3K/Akt pathway.

The results showed that MCL plays an anti-inflammatory and immunomodulatory role through NF-κB and PI3K/Akt signaling pathways upon PGN ligation.

### MCL decreases the secretion of IL-6 in sera and improves the survival status of mice challenged by *S. aureus*

**Figure 7:**
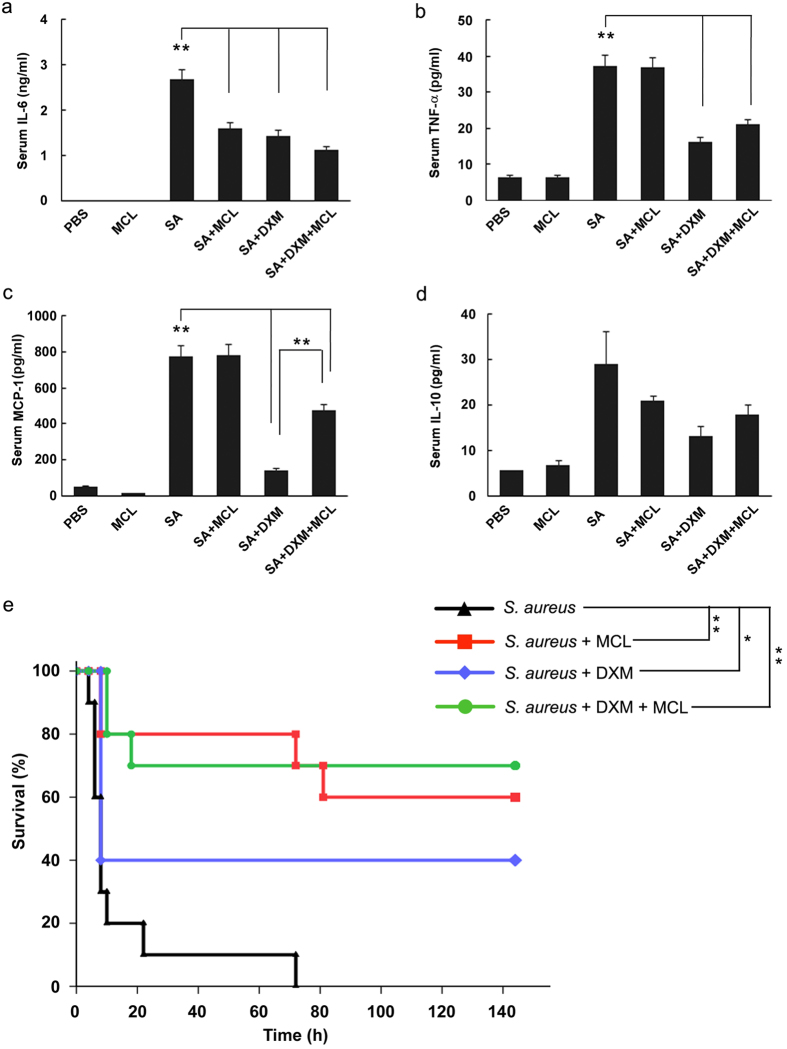
MCL reduces inflammatory mediators *in vivo* and protects mice from lethal dose of *Staphylococcus aureus* infection. The acute peritonitis mouse model was carried out in C57BL/6 J mice by intraperitoneal injection of *S. aureus* (ATCC 6538) (1 × 10^8^ CFU/mouse). Mice were divided into 6 groups (N = 6/group): PBS (0.2 mL/mouse) group, MCL (10 mg/kg) group, *S. aureus* (SA) group, SA + MCL group, SA + dexamethasone (DXM, 7 mg/kg) group, and SA + DXM + MCL group. All groups of mice were injected simultaneously with *S. aureus* and the indicated reagents. After 8 h, mice were sacrificed and blood samples were clotted for 3 h at 4 °C. Serum was collected and the concentrations of IL-6 (**a**), TNF-α (**b**), MCP-1 (**c**) and IL-10 (**d**) were measured by ELISA. Data are given as mean ± SE of 6 mice per group. **Indicates p < 0.01. C57BL/6 J mice were challenged with lethal dose of *S. aureus* (3 × 10^8^ CFU/mouse, i.p.) with different reagents, and survival statuses of different groups were recorded (**e**). Data were analyzed using Log-Rank test and survival curve was generated by Sigmaplot software. N = 10/group. *Indicates p < 0.05. **Indicates p < 0.01. SA means *S. aureus*.

Considering that MCL reduced PGN-induced inflammatory response in macrophages and DCs, we wondered whether MCL could decrease the production of inflammatory cytokines *in vivo*. We carried out *S. aureus*-infected peritonitis mouse model by intraperitoneal injection of *S. aureus* (ATCC 6538) (1 × 10^8^ CFU/mouse). Glucocorticoids (e.g. dexamethasone, DXM) are reagents to cure sepsis related diseases in the clinic^35^,^36^. In our study, DXM was chosen as a positive control in peritonitis mouse model. The dose of MCL at 10 mg/kg was selected for *in vivo* experiments, which is almost equivalent to 5 μM *in vitro*. The serum levels of cytokines showed that *S. aureus* infection dramatically increased the expression of IL-6, TNF-α, MCP-1 and IL-10 (Fig. 7a–d). The secretion of IL-6 in sera was found to be lower in MCL-treated mice compared to mice infected with *S. aureus* (Fig. 7a). However, the secretion of MCP-1, TNF-α and IL-10 was not changed significantly by MCL treatment *in vivo* (Fig. 7b–d). Meanwhile, both the clinical dose of DXM-treated group and the combined DXM and MCL-treated group showed a significant reduction in IL-6, TNF-α and MCP-1 secretion in sera.

To investigate the potential protective role of MCL against *S. aureus*-induced septic shock, we carried out a survival test. Mice were challenged by intraperitoneal injection of a lethal dose of *S. aureus* (ATCC 6538) (3 × 10^8^ CFU/mouse) and treated simultaneously with the following conditions: MCL (10 mg/kg), DXM, or the combination of DXM and MCL. PBS or MCL treatment alone did not induce any mortality within 6 days (data not shown) and caused no significant adverse effects. As shown in Fig. 7e, *S. aureus* challenge resulted in 100% lethality within 3 days. However, MCL treatment increased the survival rate to 60% (compared with *S. aureus* challenged group, p < 0.01). The results also showed that 40% of mice in the DXM treatment group survived (compared with *S. aureus* group, p < 0.05), whilst the survival rate was increased to 70% when MCL (10 mg/kg) and DXM were administered simultaneously (*S. aureus* + DXM + MCL group) (compared with *S. aureus* group, p < 0.01). Mice in the MCL-treated group or in the combined treated group exhibited significant recovery with ameliorated clinical symptoms (including lethargy, hunched posture and piloerection) compared with those in the DXM group. So, the survival data revealed that MCL treatment protected mice against septic shock induced by a lethal dose of *S. aureus*.

### MCL down-regulates IL-6, TNF-α, MCP-1 and IFN-γ secretion and ameliorates liver and kidney damage in mice infected with MRSA

MRSA induced sepsis is one of the most frequent situations in the clinic, with the common characteristics of overwhelming pro-inflammatory cytokine and chemokine production and subsequent edema, vascular leakage, vasodilatation, multiple organ failure (acute liver, lung, and kidney injury), and even death^37^.

**Figure 8:**
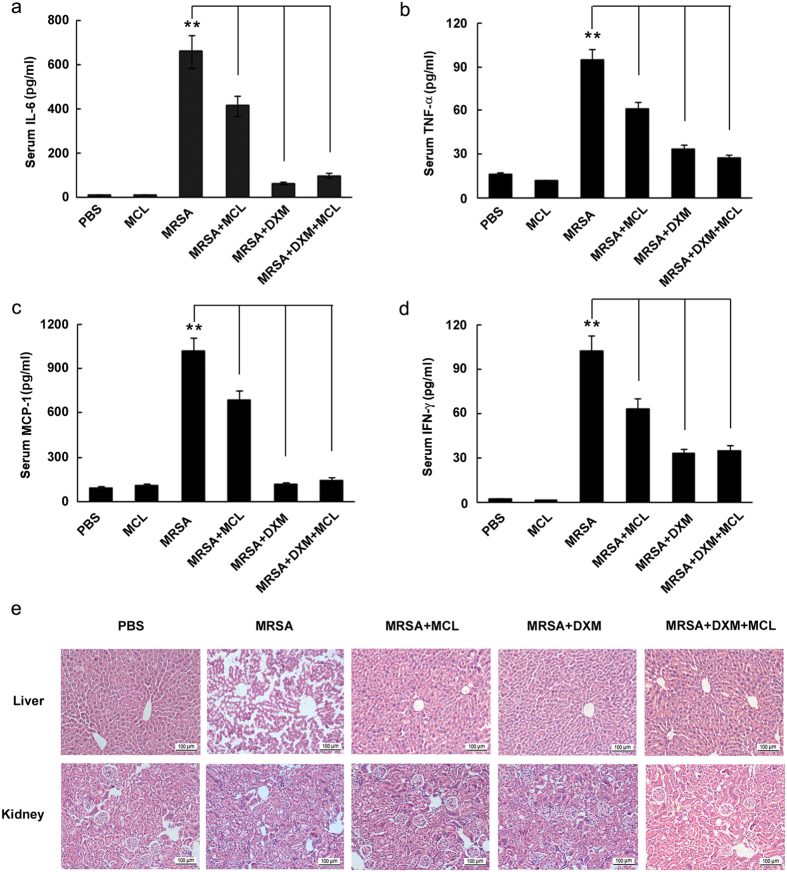
MCL inhibits the inflammatory response in mice and relieves organ damage caused by methicillin-resistant *Staphylococcus aureus* (MRSA) infection. Infective mouse model was carried out by intraperitoneal injection with MRSA (ST 239) (2 × 10^8^ CFU/mouse) in C57BL/6 J mice. Mice were divided into 6 groups (N = 5/group): PBS group, MCL (10 mg/kg) group, MRSA group, MRSA + MCL group, MRSA + dexamethasone (DXM, 7 mg/kg) group, and MRSA + DXM + MCL group. Mice were injected with MRSA and different reagents simultaneously. Twelve hours later, mice were sacrificed and blood samples were clotted and centrifuged at 4 °C. The secretion of IL-6 (**a**), TNF-α (**b**), MCP-1 (**c**) and IFN-γ (**d**) were measured by CBA method. Data are shown as mean ± SE of 5 mice per group. **Indicates p < 0.01. (**e**) H&E-staining of liver or kidney tissue sections from the indicated groups (×200). N = 3/group.

In the mouse model of MRSA infection, MCL was found to play an anti-inflammatory role of down-regulating the secretion of inflammatory cytokines in sera. The concentrations of IL-6, TNF-α, MCP-1, and IFN-γ in sera were decreased by MCL treatment (Fig. 8a–d). The DXM-treated group and the combined DXM and MCL treated group also showed significantly reduced cytokine secretion.

The histopathological damage of liver and kidney was investigated. H&E-stained livers in phosphate-buffered saline (PBS)-treated group exhibited a normal histological structure without pathological changes (Fig. 8e). Upon MRSA infection (2 × 10^8^ CFU/mouse), there were significant inflammatory manifestations, including inflammatory cell infiltration, necrotic hepatic cells and disordered array of hepatic plates. Compared with MRSA-treated group, the pathological changes of livers in mice treated with MCL, DXM or their combination exhibited milder cellular injury and less inflammatory cell infiltration. Similar to the inflammatory manifestations in livers, kidneys from mice treated with MCL, DXM, or their combination exhibited less severe pathological changes. The results demonstrated that MCL as well as DXM attenuated the pathological changes of liver and kidney during MRSA infection.

## Discussion

*S. aureus*, the main pathogen of G^+^ bacteria, is one of the most prevalent causes of sepsis with high mortality in U.S. hospitals^1^,^7^,^8^. Along with the emergence of MRSA, patients with sepsis face an increased risk of death^10^,^11^. A number of therapeutic strategies were put forward to aim at treating sepsis induced by *S. aureus* or MRSA targeting either the pathogens or the host’s response.

Some advances were achieved to treat *S. aureus* or MRSA infection by targeting the pathogens. For instance, a monoclonal antibody which targets *S. aureus* surface protein A (SasA), 2H7, was shown to promote the phagocytosis of neutrophils to kill *S. aureus* in the bloodstream^38^. The novel antibody-antibiotic conjugate (AAC), which is made up of an anti-*S. aureus* antibody and a highly efficacious antibiotic, can be taken up by the host’s cells and eliminate intracellular *S. aureus*^39^. A different therapeutic approach targets the formation of MRSA biofilm to inhibit MRSA-triggered sepsis. ND8008, a novel NO-releasing derivative of dexamethasone, may be another option for the treatment of sepsis caused by MRSA^10^.

In the clinic, sepsis is usually accompanied by SIRS, which is characterised by over-expression of inflammatory cytokines (IL-6, TNF-α, IL-1β, IFN-γ, etc.) and anti-inflammatory cytokines (IL-10, TGF-β and IL-1Ra)^40^,^41^ and subsequent dysfunction of various organs^7^,^42^. Both TNF-α and IL-1β are released from macrophages quickly after the inciting event. They act synergistically for the development of fever and to induce septic shock by vascular permeability, hemorrhage and severe pulmonary edema^43^. Subsequently, IL-6 concentrations are increased and plasma levels remain stably elevated during the occurrence of multiple organ failure and septic shock^44^, as well as mortality^45^. IFN-γ is a cytokine mainly produced by activated NK cells, cytotoxic T cells and Th1 cells^46^. The neutralization of IFN-γ or the blocking of its receptor makes mice more resistant to an LPS-induced septic shock^47^,^48^. The role of IL-10 in inflammatory diseases is controversial. Although it is a well-known anti-inflammatory cytokine, high level of IL-10 has been reported to be associated with high mortality in severe abdominal sepsis. In the early stage (within 24 h) of septic patients who died, serum levels of IL-10 were found to be elevated^29^. Extremely high expression of pro-inflammatory cytokines as well as anti-inflammatory cytokines makes the environment of the host like a smoking battlefield in sepsis. Thus, the other therapeutic strategy against sepsis was brought forth to aim at the regulation of immune response of the host.

As we reported earlier, a small compound ephedrine hydrochloride (EH) from ephedrine (one of the main active components of *Ephedra sinica*, also known in Chinese as Ma Huang) maintains the balance of pro-inflammatory and anti-inflammatory cytokine production in response to LPS or PGN, which makes it a promising candidate for the treatment of inflammatory diseases^13^,^49^. Our recent work demonstrated that MCL plays a protective role in LPS-induced septic shock^29^. In this study, we showed that MCL inhibited PGN-evoked expression of inflammatory cytokines, such as IL-6, TNF-α, IL-1β, MCP-1 and IL-10 in Raw264.7, primary macrophages and BMDCs. Consistently, MCL also inhibited production of IL-6, TNF-α, MCP-1 and IL-10 in human THP-1 monocytes.

Although excessive inflammatory response could be detrimental to the host, a proper inflammatory response is essential for eradicating infectious microorganisms^20^. The *in vivo* experiments showed that MCL decreased IL-6 secretion in sera and protected mice from lethal *S. aureus* challenge. MCP-1/CCL2 is a potent chemotactic factor for monocytes/macrophages and dendritic cells to the sites of inflammation caused by infection or tissue injury. Inhibition of CCL2-CCR2 signaling blocks the recruitment of inflammatory monocytes^50^. MCP-1/CCL2 and its receptor (CCR2) knockout mice displayed a decrease in macrophage recruitment and inflammation at sensor implantation sites compared to normal mice^51^. The recruitment of monocytes/macrophages is protective in an early stage of bacterial infection, promoting bacterial uptake and restriction of bacterial colonization^20^. As reported, the colonization of *Streptococcus pneumoniae* is gradually cleared through phagocytosis by monocytes/macrophages, which are recruited to sites of infection through their expression of the chemokine receptor CCR2 and correlated with its ligand MCP-1/CCL2^20^. Though both of DXM treatment group and combinational treatment group (DXM + MCL) decreased *S. aureus* induced MCP-1 level at 8 h compared with model group (*S. aureus* only) in lethal peritonitis model (Fig. 7), the level of MCP-1 in the combinational treatment group was maintained significantly higher than that of DXM treatment group (p = 0.0021) (Fig. 7c). High level of MCP-1 recruits monocytes/macrophages to sites of infection and may facilitate early clearance of *S. aureus*, which may partially interpret the slightly better therapeutic role (though not statistically different) of combinational group than DXM treatment group. In the peritonitis mouse model induced by intraperitoneal injection of MRSA, MCL also suppressed the over-expression of inflammatory cytokines (IL-6, TNF-α, MCP-1 or IFN-γ) and relieved organ damage of liver and kidney. Thus, MCL plays a protective role against sepsis induced by *S. aureus* and MRSA likely through down-regulating the expression of different cytokines. MCL calms the excessive, tissue-damaging inflammation down to maintain the homeostasis of the host by inhibiting the expression of both pro-inflammatory and anti-inflammatory cytokines.

*S. aureus* and its derivative PGN are recognized by TLR2 on the cell surface, and by NOD2 in the cytosol, leading to the activation of subsequent common signaling pathways (NF-κB and MAPKs), accounting for the induction of pro-inflammatory cytokines by innate immune cells, including macrophages, dendritic cells and neutrophils^13^. PI3K/Akt signaling pathway, together with NF-κB and MAPK pathways, plays an important role in PGN-induced production of pro-inflammatory cytokines, chemokines, etc^52^. As recently reported, NOD2 recognizes lysozyme-digested PGN processed by professional phagocytes and releases chemokine MCP-1/CCL2 to recruit additional monocytes/macrophages to restrict/clear *Streptococcus pneumonia* colonization in mice^20^. NOD2 also plays a specific protective role against intracellular bacterial infection in the intestine (e.g., *Listeria Monocytogenes*). Interestingly, NOD2-deficient mice are susceptible to *Listeria Monocytogenes* infection via the oral route, but not through intravenous or peritoneal delivery^53^. In this study, peritonitis mouse model was induced by intraperitoneal injection of extracellular bacteria *S. aureus* or MRSA. Accroding to the character of extracellular infection about *S. aureus* and the limited delivery pathway of PGN from G^+^ bacteria into the cytosol (mainly through phagocytosis)^14^, TLR2 should play a more important sensing role than NOD2 in the recognition of PGN from *S. aureus*.

Here, we demonstrated that MCL inhibited PGN-induced phosphorylation of IκBα (Ser32/36) (Fig. 5b) and subsequent activation of NF-κB luciferase reporter gene (Fig. 5c) in macrophages, accounting for the decreased secretion of inflammatory cytokines. MCL decreased PGN-mediated IL-6 secretion likely through down-regulating NF-κB activation (Fig. 6c). Upon PGN/TLR2 ligation, IL-10 expression is regulated by several signaling pathways in macrophages, including ERK, p38, NF-κB and PI3K/Akt^19^. In mouse peritoneal macrophages, EH increases PGN-induced phophorylation of Akt (Thr308) and pretreatment of LY294002, PI3K inhibitor, abrogated the enhanced IL-10 expression and the decreased IL-6 expression by EH with the stimulation of PGN^15^. In the current study, MCL was also shown to inhibit PGN-induced phosphorylation of Akt (Thr308) and the downstream p70S6K (Thr389) in mouse primary peritoneal macrophages (Fig. 6a). Over-expression and activation of Akt increased PGN-induced IL-10 secretion but MCL abolished such an increase by inhibiting PI3K/Akt pathway (Fig. 6d). It indicated MCL inhibited PGN-mediated IL-10 expression at least in part through PI3K/Akt. Because NF-κB is a well-known transcriptional factor to elicit IL-10 expression in macrophages and DCs after TLR ligation^19^, the potential contributing role to IL-10 expression by MCL-inhibited NF-κB pathway could not be excluded. Another possible mechanism of NF-kB activation promoted by PI3K pathway was reported during *S. aureus* infection. Infection of *S. aureus* causes the recruitment of active Rho GTPases Rac1 and PI3K to the TLR2 cytosolic domain. Dominant-negative Rac1N17 blocks NF-κB transactivation. A signaling cascade composed of TLR2, Rac1, PI3K and Akt targets nuclear p65 translocation in I-κB-independent manner and is essential in innate immune cells^54^. MCL decreased PI3K/Akt activation might contribute to a decreased NF-κB activation and subsequent down-regulation of IL-10.

In summary, we showed that MCL has potential anti-inflammatory and immunosuppressive activities in the treatment of sepsis and septic shock caused by *S. aureus* and MRSA. More efforts should be put into studying the exact molecular targets of MCL and investigating the combined role of MCL with antibiotics or glucocorticoids in the treatment of *S. aureus* or MRSA-induced sepsis clinically. Considering MCL appears to modulate inflammation caused by PGN and LPS (the main PAMPs of G^+^ and G^−^ bacteria, respectively)^29^, as well as *S. aureus* and MRSA, its use as a universal therapeutic agent against bacterial infection should be thoroughly addressed, especially in the context of infection caused by antibiotic resistant bacteria.

## Materials and Methods

### Mice and reagents

Female C57BL/6 J mice (age 4 to 8 weeks, weight 20 ± 3 g) were purchased from Joint Ventures Sipper BK Experimental Animal Co. (Shanghai, China) and acclimated for at least 1 week before use. All mice were housed in an SPF colony with a 12 h light/dark cycle. All animal studies were performed in accordance with the National Institute of Health Guide for the Care and Use of Laboratory Animals, with the approval of the Scientific Investigation Board of Shanghai University of Traditional Chinese Medicine (Shanghai, China). Anti-β-Actin antibody, and horseradish peroxidase-coupled secondary antibodies were purchased from Santa Cruz Biotechnology (Santa Cruz, CA). Phospho-antibodies against the extracellular signal-regulated kinase 1/2 (ERK1/2, Thr202/Tyr204), c-Jun N-terminal kinase/stress-associated protein kinase (JNK, Thr183/Tyr185), p38 MAPK (Thr180/Tyr182), Akt (Ser473), Akt (Thr308), p70S6K (Thr389), 4E-BP1 (Thr37/46), IκBα (Ser32/36) and corresponding antibodies against total proteins were from Cell Signaling Technology (Beverly, MA). PGN from *S. aureus*, DMSO, phorbol-12-myristate-13-acetate (PMA) and Dexamethasone (DXM) (Molecular Formula: C_22_H_29_FO_5_; Molecular Weight: 392) were obtained from Sigma (St. Louis, MO). Micheliolide (MCL) (Molecular Weight: 248.3, purity >99%, chemical structure shown in Fig. 1a; purity shown in Suppl. Fig. 1) was isolated from the *Michelia compressa* (Magnoliaceae). For the following experiments, MCL was dissolved in DMSO at a concentration of 40 mM as a stock solution and diluted to the indicated concentrations with medium in advance. Recombinant mouse granulocyte-macrophage colony stimulating factor (GM-CSF) and IL-4 were purchased from R&D Systems (Minneapolis, MN).

### Bacterial strains and growth conditions

*Staphylococcus aureus (S. aureus*) strain type ATCC 6538 and methicillin-resistant *Staphylococcus aureus* (MRSA) strain type ST239 were obtained from ATCC (Manassas, VA). *S. aureus* and MRSA cultures were grown overnight at 37 °C in Luria-Bertani (1% Tryptone, 0.5% Yeast extract, 1% NaCl) broth. *S. aureus* and MRSA bacteria were collected and re-suspended in sterile phosphate buffered saline (PBS).

### Cell culture

Mouse macrophage-like cell line Raw264.7 was obtained from ATCC (Manassas, VA) and cultured as described previously^29^. Human acute monocytic leukemia cell line THP-1 was obtained from ATCC (Manassas, VA) and was grown in RPMI-1640 supplemented with 10% heat-inactivated FBS. Thioglycolate-elicited mouse primary peritoneal macrophages were prepared from female C57BL/6 J mice (6–8 weeks of age) as described previously^29^. After 2 h, non-adherent cells were removed and the adherent cells were used as peritoneal macrophages. Bone marrow-derived dendritic cells (DCs) from C57BL/6 J mice (4 weeks of age) were generated as described^49^.

### Detection of cell apoptosis by flow cytometry assay

Mouse primary peritoneal macrophages were treated with indicated concentrations of MCL for 18 h in the presence or absence of PGN (25 mg/L), and then were harvested and labeled with PE-Annexin V and 7-Amimo-Actinomycin (7-AAD) provided by BD Pharmingen (San Diego, USA), following the manufacturer’s instructions. Samples were examined by a flow cytometer BD Accuri^TM^ C6 (BD, San Jose, USA). Data were analyzed using CFlow software (BD, San Jose, USA).

### Detection of cytokine production

Enzyme-linked immunosorbent assay (ELISA) kits for murine IL-6, TNF-α, MCP-1 and IL-10 were purchased from R&D Systems (Minneapolis, MN). IL-6, TNF-α, MCP-1 and IL-10 in culture supernatants were measured by ELISA according to the manufacturer’sinstructions^15^.

### RNA quantification

Total RNA was prepared from cells using TRIzol reagent (Invitrogen, Carlsbad, CA) according to the manufacturer’s instructions. Complementary DNA (cDNA) was synthesized from 0.5 μg total RNA by reverse transcriptase (Takara, Dalian, China). Quantitative real-time RT-PCR (qRT-PCR) analysis was performed with the SYBR RT-PCR Kit (Takara, Dalian, China) and LightCycler (Roche Diagnostics, Indianapolis, IN) as described previously^35^. Primers used for qRT-PCR amplification of β-Actin, and IL-1β were described previously^35^. Data were normalized by the level of β-Actin expression in each sample.

### Western blot analysis

Cells were lysed with M-PER^TM^ Protein Extraction Reagent (Pierce, Rockford, IL) supplemented with protease inhibitor cocktail and phosphatase inhibitor cocktail (Roche, Basel, CH). The protein concentration of each sample was measured with BCA assay (Pierce, Rockford, IL). Equal amounts of extracts were separated by 10% SDS-PAGE, transferred onto a PVDF membrane, and then blotted as described previously^15^. Actin was used as an internal control.

### Plasmid constructs and transfection

Mouse NF-κB luciferase reporter gene plasmid and pRL-TK-*Renilla*-luciferase plasmid were described previously^35^. Transfection of Raw264.7 macrophages with jetPEI^TM^ (PolyPlus-Transfection, Illkirch, France) was performed according to the manufacturer’s instructions. Akt expressing plasmid (Myr-Akt-HA) and the corresponding empty vector plasmid were kind gifts from Prof. Chaofeng Han (National Key Laboratory of Medical Immunology, Second Military Medical University).

### Assay of luciferase reporter gene expression

Raw264.7 was cotransfected with the mixture of 100 ng NF-κB luciferase reporter plasmid and 20 ng pRL-TK-*Renilla*-luciferase plasmidusing jetPEI^TM^ (Polyplus). After 30 h, cells were stimulated with PGN for 24 h, and luciferase activities were measured with Dual-Luciferase Reporter Assay System (Promega) according to the manufacturer’s instructions. To exclude the influence of transfection efficiency, data were normalized by division of *Firefly* luciferase activity with that of *Renilla* luciferase. The relative values were presented as fold increase^35^.

### *In vivo S. aureus* or MRSA challenge and serum cytokine detection

Female C57BL/6 J mice (6–8 weeks old) were injected intraperitoneally with *S. aureus* (ATCC 6538) (1 × 10^8^ CFU/mouse) or MRSA (ST 239) (2 × 10^8^ CFU/mouse) with or without MCL or DXM. *S. aureus* and MRSA bacteria were prepared in sterile phosphate buffered saline (PBS) in advance. Mice were killed 8 h or 12 h after injection of *S. aureus* or MRSA respectively. The plasma samples of *S. aureus* infection were clotted and collected for cytokine analysis by ELISA.

The plasma samples of MRSA infection were clotted and collected for cytokine analysis by cytometric beads array (CBA) mouse inflammation kit (BD Biosciences, San Jose, USA) according to the manufacturer’s instructions. In brief, 50 μL mouse serum was added to a mixture of capture beads coated with mAb to a panel of cytokines (IL-6, IL-10, MCP-1, TNF-α, IFN-γ and IL-12p70) and a mouse inflammation PE detection reagent. After 2 h incubation, the capture beads were washed and acquired on a flow cytometer BD Accuri^TM^ C6 (BD, San Jose, USA), and then analyzed with BD FCAP Array Software (BD Biosciences, San Jose, USA).

PBS or DXM (7 mg/kg, equivalent to the clinical dose) were used as a negative or positive control, separately^15^. The synergistic role of MCL and DXM combination was verified *in vivo*.

### Histopathology

Female C57BL/6 J mice (6–8 weeks old) were injected intraperitoneally with MRSA (ST 239) (2 × 10^8^ CFU/mouse), MCL or DXM as indicated. After 12 h, mice were sacrificed and liver and kidney tissues were collected to be fixed with 4% formaldehyde and paraffin-embedded. The tissues were sliced and stained with hematoxylin and eosin (H&E). The histopathological changes were observed using a Zeiss Imager M2 microscope (Carl Zeiss Micro Imaging) equipped with an Axio CamHRc CCD camera (Carl Zeiss).

### *S. aureus*-induced septic shock mouse model

The septic shock mouse model was established by intraperitoneal injection of *S. aureus* (ATCC 6538) (3 × 10^8^ CFU/mouse). The experiments were carried out in the following groups: *S. aureus* treatment group; *S. aureus* + MCL (10 mg/kg) group; *S. aureus* + DXM (7 mg/kg) group and the combined treatment *S. aureus* + DXM + MCL (10 mg/kg) group. The survival status was recorded over time as previously described^50^.

### Statistical analysis

Results were given as means ± standard deviation (SD) or means ± standard error (SE). Comparisons between 2 groups were done using Student’s *t* test analysis. Survival analysis between multiple groups were done using Log-Rank test. The survival curve was drawn by Sigmaplot software. Statistical significance was determined as p < 0.05 or p < 0.01.

## Additional Information

**How to cite this article:** Jiang, X. *et al*. Micheliolide provides protection of mice against *Staphylococcus aureus* and MRSA infection by down-regulating inflammatory response. *Sci. Rep.* **7**, 41964; doi: 10.1038/srep41964 (2017).

**Publisher's note:** Springer Nature remains neutral with regard to jurisdictional claims in published maps and institutional affiliations.

## Supplementary Material

Supplementary Material
