# Supplementary material for: Micheliolide provides protection of mice against Staphylococcus aureus and MRSA infection by down-regulating inflammatory response
Source: Sci Rep. 2017 Feb 6;7:41964. doi: 10.1038/srep41964 (PMC5292736; doi:10.1038/srep41964)
Supplement: Supplementary Material [file srep41964-s1.doc]

**SUPPLEMENTARY MATERIAL**

**Micheliolide provides protection of mice against *Staphylococcus aureus* and MRSA infection by down-regulating inflammatory response**

Xinru Jiang^1^, ^#^, Yuli Wang^1, #^, Yifei Qin^2, #^, Weigang He^1^, Adel Benlahrech^3^, Qingwen Zhang^1^, Xin Jiang^1^, Zhenhui Lu^4^, Guang Ji^5, *^, Yuejuan Zheng^1^, ^3, *^

^1^ Department of Immunology and Microbiology, Shanghai University of Traditional Chinese Medicine, Shanghai 201203, China;

^2^ School of Clinical Medicine, Shaanxi University of Chinese Medicine, Xi xian New District, Shaanxi 712046, China;

^3^ MRC Human Immunology Unit, Weatherall Institute of Molecular Medicine, Nuffield Department of Medicine, University of Oxford, Oxford OX3 9DS, UK;

^4^ Department of Respiration, Longhua Hospital, Shanghai University of Traditional Chinese Medicine, Shanghai 200032, China;

^5^ Institute of Digestive Diseases, China-Canada Center of Research for Digestive Diseases (ccCRDD), Longhua　Hospital, Shanghai University of Traditional Chinese Medicine, Shanghai 200032, China;

^#^ These authors contributed equally to this work. Correspondence and requests for materials should be addressed to Y.-J.Z. (email: 13641776412@163.com or zhengyj@shutcm.edu.cn) or G. J. (email: jiliver@vip.sina.com).

**Supplementary Figure S1**


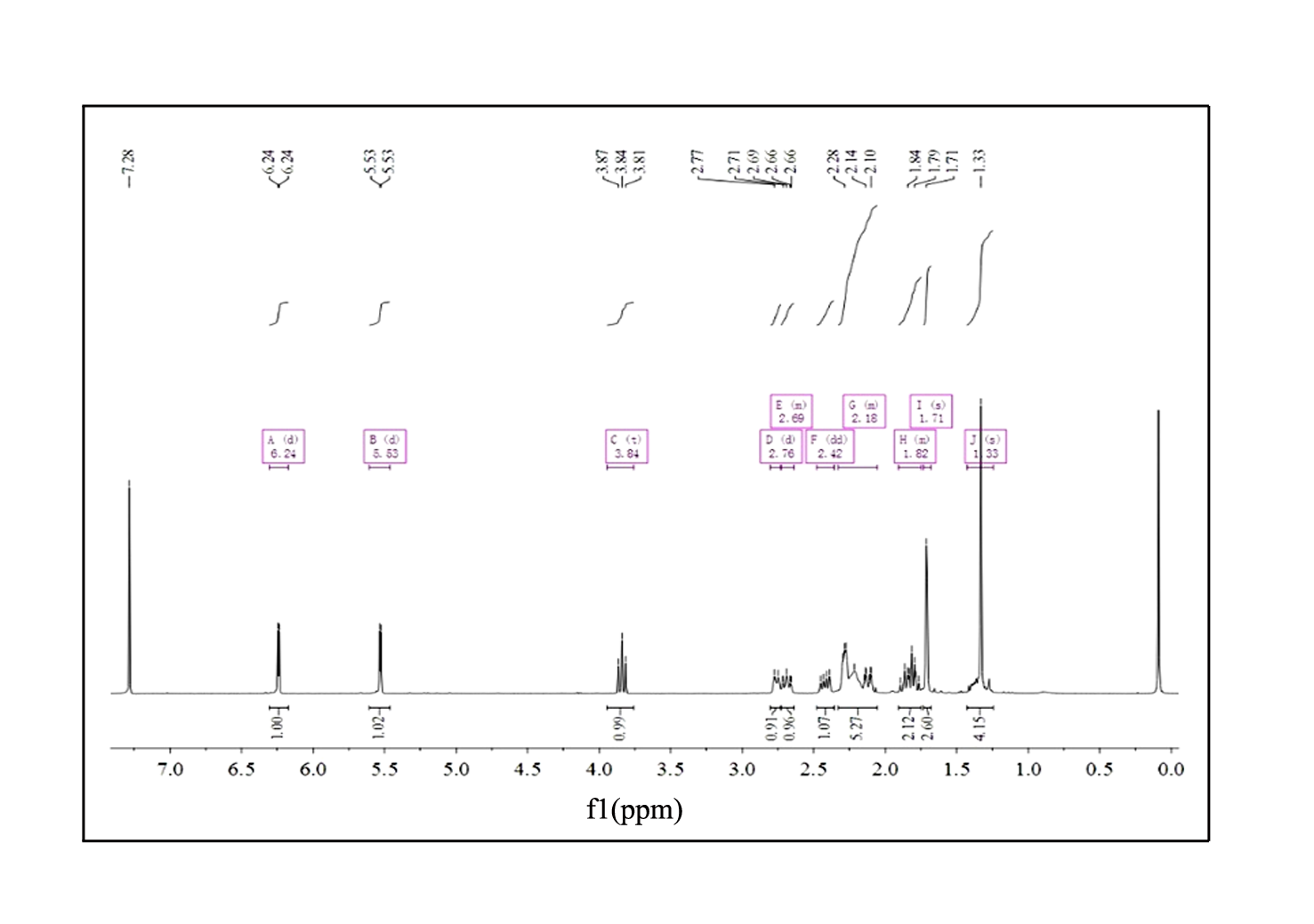


**Figure S1. The HNMR spectrum of micheliolide.** Micheliolide (MCL) is a sesquiterpene lactone (Fig. 1A), isolated from *Michelia compressa* (Magnoliaceae) according to the method described^1^.We are convinced that the purity of MCL can reach 99% by HNMR spectrum. HNMR characteristic spectrum and data are shown as the following: 1H NMR (400 MHz, CDCl3) δ 6.24 (d, J = 3.3 Hz, 1H), 5.53 (d, J = 3.0 Hz, 1H), 3.84 (t, J = 10.3 Hz, 1H), 2.76 (d, J = 10.5 Hz, 1H), 2.73 – 2.64 (m, 1H), 2.42 (dd, J = 16.4, 8.4 Hz, 1H), 2.33 – 2.05 (m, 5H), 1.91 – 1.75 (m, 2H), 1.71 (s, 3H), 1.33 (s, 4H).
